# CDC’s Model Aquatic Health Code, First Edition

**Published:** 2014-09-05

The first edition of the Model Aquatic Health Code (MAHC) was released on August 29, 2014, and is now available from CDC online at http://www.cdc.gov/mahc. The MAHC is a guidance document that jurisdictions can use to update or implement codes, rules, regulations, guidance, laws, or standards governing swimming pools, spas, hot tubs, and other public, treated, recreational water venues to reduce infectious disease outbreaks, drowning, and chemical injuries.

In the United States, no federal agency regulates the design, construction, operation, and maintenance of public swimming pools and other public, treated, recreational water venues. All pool codes are independently written and enforced by state and/or local agencies. In 2005, local, state, and federal public health officials and representatives of the aquatic sector requested that CDC develop a model, evidence-based code. Since 2007, CDC has led a national collaborative effort with public health, industry, and academic representatives from across the United States to develop the MAHC.

CDC will work with national partners to periodically update the MAHC to ensure it stays current with the latest industry advances and public health findings. The Conference for the Model Aquatic Health Code (CMAHC) (http://www.cmahc.org) is a nonprofit organization created in 2013 to support and improve public health by promoting healthy and safe aquatic experiences for everyone. CMAHC members will suggest MAHC revisions for CDC’s final determination.

